# Highlight selection of radiochemistry and radiopharmacy developments by editorial board

**DOI:** 10.1186/s41181-024-00268-w

**Published:** 2024-05-16

**Authors:** Jun Toyohara, Danielle Vugts, Oliver C. Kiss, Sergio Todde, Xiang-Guo Li, Zhibo Liu, Zhi Yang, Nic Gillings, Emiliano Cazzola, Wiktor Szymanski, Nick van der Meulen, Raymond Reilly, Carlotta Taddei, Ralf Schirrmacher, Zijing Li, Yohannes Jorge Lagebo, Naoual Bentaleb, Marta de Souza Albernaz, Suzanne Lapi, Caterina Ramogida, Archana Mukherjee, Javier Ajenjo, Winnie Deuther-Conrad, Cécile Bourdeau

**Affiliations:** 1 Tokyo Metropolitan Institute for Geriatrics and Gerontology, Tokyo, Japan; 2 Amsterdam University Medical Center, Amsterdam, The Netherlands; 3 Institute of Radiopharmaceutical Cancer Research, Helmholtz-Zentrum Dresden-Rossendorf (HZDR), Dresden, Germany; 4 University of Milano-Bicoccia, Tecnomed Foundation, Monza, Italy; 5 Turku PET Centre and Department of Chemistry, and InFLAMES Research Flagship Center, University of Turku, Turku, Finland; 6 Peking University, Beijing, China; 7 Peking University Cancer Hospital & Institute, Beijing, China; 8 Copenhagen University Rigshospitalet, Copenhagen, Denmark; 9 Sacro Cuore Hospital, Verona, Italy; 10 University of Groningen, Groningen, The Netherlands; 11 Paul Scherrer Institute (PSI), Villigen, Switzerland; 12 University of Toronto, Toronto, Canada; 13 Life Molecular Imaging GmbH, Berlin, Germany; 14 University of Alberta, Edmonton, Canada; 15 Xiamen University, Xiamen, China; 16 Addis Ababa University, Addis Ababa, Ethiopia; 17 National Center for Nuclear Energy, Science and Technology-CNESTEN, Rabat, Morocco; 18 University Hospital Clementino Fraga Filho, Federal University of Rio de Janeiro, Rio de Janeiro, Brazil; 19 University of Alabama at Birmingham, Birmingham, USA; 20 Simon Fraser University, Burnaby, Vancouver, Canada; 21 TRIUMF, Burnaby, Vancouver, Canada; 22 Bhabha Atomic Research Center and Homi Bhabha National Institute, Mumbai, India; 23 Molecular Imaging Program at Stanford (MIPS), Dept of Radiology, School of Medicine, Stanford University, Stanford, CA) USA; 24 GIP Arronax, Nantes, France; 25 Institute of Radiopharmaceutical Cancer Research, Helmholtz-Zentrum Dresden-Rossendorf (HZDR), Leipzig, Germany

**Keywords:** Highlight articles, Radiochemistry, Radiopharmacy, Radiopharmaceutical sciences, Nuclear medicine, Trends in radiopharmaceutical sciences

## Abstract

**Background:**

The Editorial Board of EJNMMI Radiopharmacy and Chemistry releases a biannual highlight commentary to update the readership on trends in the field of radiopharmaceutical development.

**Main body:**

This selection of highlights provides commentary on 24 different topics selected by each coauthoring Editorial Board member addressing a variety of aspects ranging from novel radiochemistry to first-in-human application of novel radiopharmaceuticals.

**Conclusion:**

Trends in radiochemistry and radiopharmacy are highlighted. Hot topics cover the entire scope of EJNMMI Radiopharmacy and Chemistry, demonstrating the progress in the research field in many aspects.

## Background

Each individual coauthoring member of the Editorial Board has selected to highlight an article that has appeared in the radiochemistry, radiopharmacy and imaging agent literature during the period July-December 2023. The aim of this collaborative initiative is to create a biyearly overview for the readers summarizing the latest trends and hot topics in the field.

### Modulation of enhanced permeation and retention effects of radioisotope-encapsulated liposomes using coordination chemistry

#### By Jun Toyohara

**Fig. 1:**
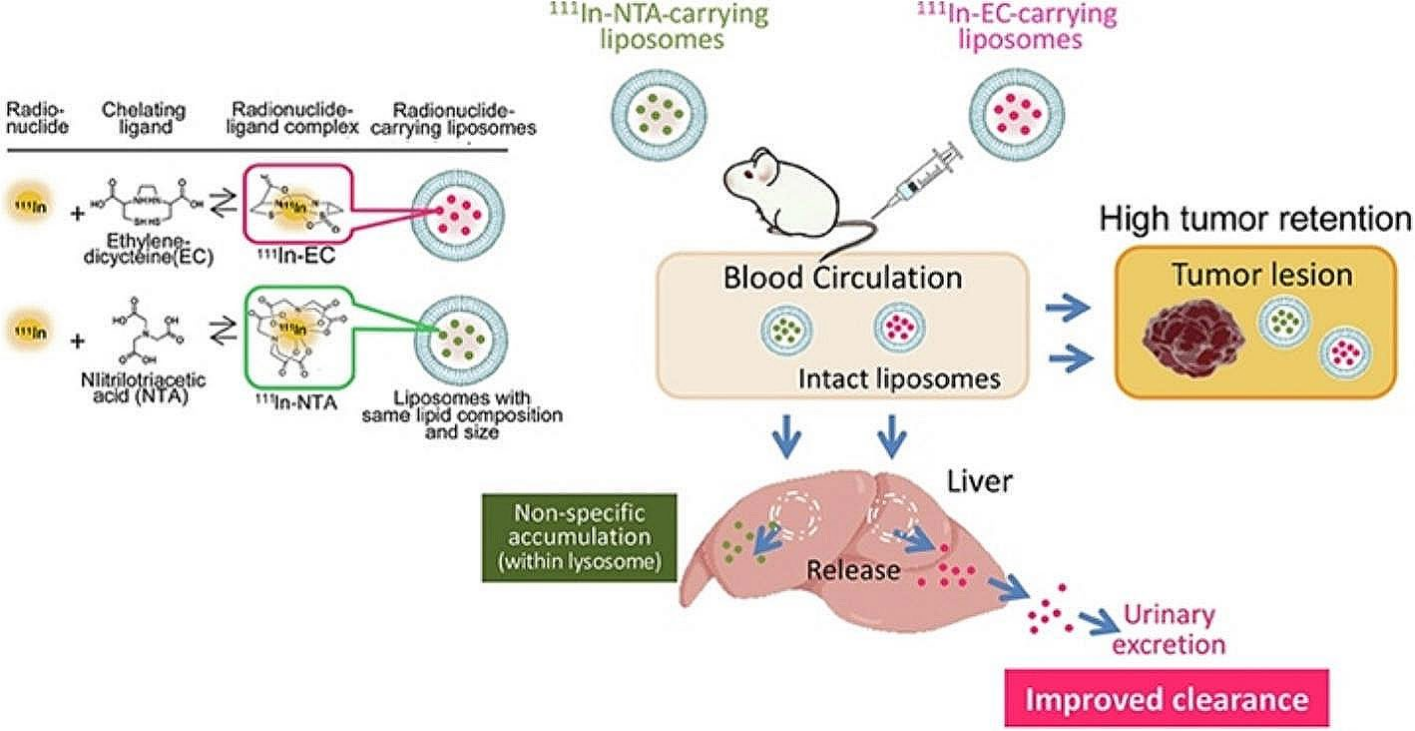
New liposome-radionuclide-chelate combination for targeting tumors and achieving rapid clearance from healthy tissue. The graphical abstract is reproduced with permission (Umeda et al. 2023)

Vascular permeability in tumor tissue is significantly increased compared to normal tissue, and macromolecules and microparticles easily leak out from blood vessels within tumors. On the contrary, because the lymphatic system is not developed within tumors, substances that reach the tumor tissue by this system are retained. This characteristic is called the enhanced permeation and retention (EPR) effect and is the basis for passive targeting of tumor tissues using macromolecules such as liposomes, nanoparticles, polymerized drugs etc. A drawback of utilizing the EPR effect in this way in radioisotope-encapsulated liposomes is that a large amount of liposomes accumulates in the reticuloendothelial system, such as the liver and spleen. To address this problem, a new approach has been developed to reduce radioactivity in the reticuloendothelial system by rapidly excreting the radioactivity from these tissues (Umeda et al. 2023). They have developed a method to significantly reduce the retention of radioactivity in the reticuloendothelial system and selectively deliver therapeutic radionuclides only to cancer tissues (Fig. 1). Specifically, when a ^111^In-ethylene-dicystein complex was encapsulated in liposomes, radioactivity accumulation in the liver and spleen was reduced without impairing tumor accumulation. However, when nitrilotriacetic acid was used as a ligand, radioactivity continued to remain in the liver and spleen. In tumor tissues, liposomes were relatively stable and radioactivity was retained within the liposomes. In contrast, liposomes in the liver were rapidly degraded, and the encapsulated radioisotope-complex was released, followed by capture of the conventional nitriloacetic acid complex in the lysosomal fraction, whereas the rapid release of the ethylene-dicystein from the cells owing to uncaptured in the lysosome and its hydrophilic properties.

### Are photocatalyzed reactions with [^11^C]CO_2_ leading to useful PET tracers?

#### By Danielle Vugts

**Fig. 2:**
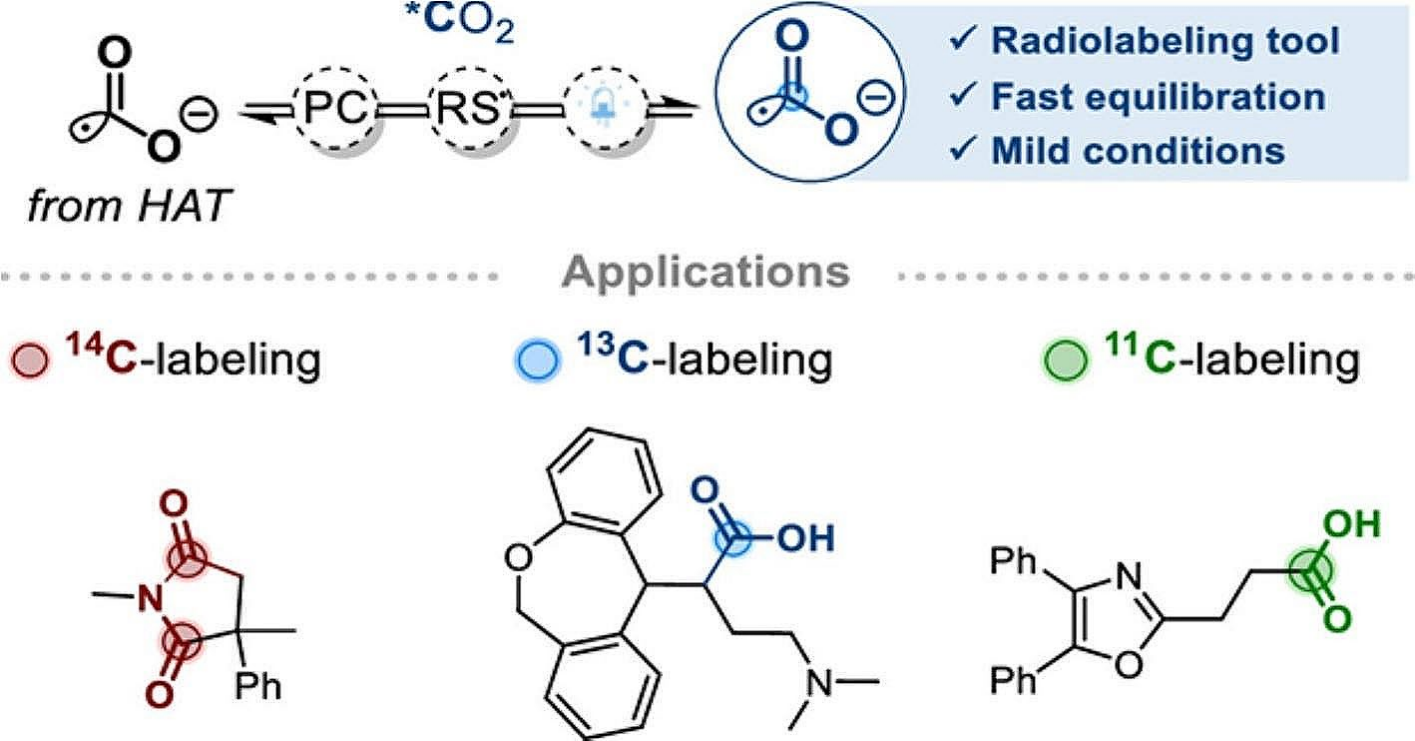
Reprinted with permission (Malandain et al. 2023). Copyright American Chemical Society

There is a continuous interest in developing new radiolabeling methods to expand the radiochemical space. With the emerging long axial field-of-view (LAFOV) PET/CT cameras the interest in ^11^C-radiochemistry is increasing. These LAFOV scanners have an increased sensitivity and therefore less radioactivity and/or lower molar activity tracers can be injected, while still getting high quality images. Using [^11^C]CO_2_, directly obtained from the cyclotron, for PET tracer synthesis sounds ideal, but is it as easy as it sounds? The relatively low reactivity and potentially low molar activity, due to isotopic dilution with atmospheric CO_2_, do not help. However, an interesting new method for carbon isotope radiolabeling which is based on a fast equilibrium between radical anion [^12^C]CO_2_^•−^ and ^11^C, ^13^C and ^14^C-labeled CO_2_ has been reported (Fig. 2) (Malandain et al. 2023). Initial experiments were done using [^13^C]CO_2_ and demonstrated that the reaction of potassium formate ([^12^C]HCOOK) and [^13^C]CO_2_ under mild photocatalytic conditions resulted in [^13^C]HCOOK in good isotopic enrichment. Using this method not only formate labeling could be achieved, but also ^13^C-hydrocarboxylation via reaction of [^13^C]CO_2_ with styrenes, acrylamides or enones. The authors also demonstrated the applicability to [^11^C]CO_2_ chemistry by performing ^11^C-hydroxycarboxylation. Good radiochemical conversions of around 35% could be obtained in 10 min under mild reaction conditions. Furthermore, they synthesized [^11^C]oxaprozin in a decent radiochemical conversion of 15%, but low molar activity (74 MBq/µmol) which is expected for such type of chemistry. Despite the fact that the molar activity is very low, making this method not suited for PET tracers targeting a low abundant receptor, it demonstrates the possibilities of photocatalyzed [^11^C]CO_2_ chemistry and calls for further improvements.

### The „whole“ in radiopharmacy: from ”the origin” to “theragnostic”

#### By Oliver C. Kiss

**Fig. 3:**
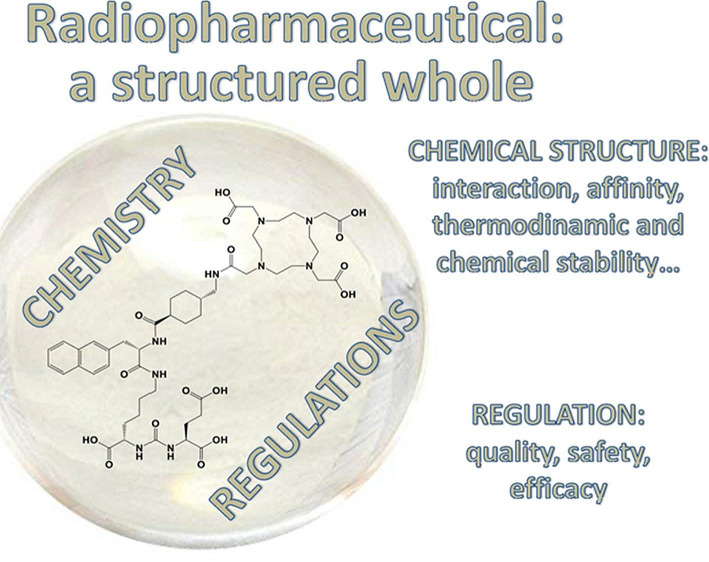
Reprinted with permission (Boschi and Todde 2021)

Few scientists in nuclear medicine and/or radiopharmacy have a history in philosophy prior to their academic careers or post their academic careers (Haberkorn and Czernin 2019). One of the latter scientists, Stefano Boschi from Bologna/Italy, has started writing editorials together with friends and colleagues about the harmony and similarities between radiopharmacy and philosophy by showing us the transformation of radiotracers into radiopharmaceuticals as “structured wholes” with chemistry and regulations being two balanced components (Fig. 3) (Boschi and Todde 2021). This concept was then further expanded into how the clinical need for radiopharmaceuticals could and regulatory aspects should be weighed to fulfil the ultimate goal: patient benefit (Boschi and Todde 2023). In his third and hopefully not last editorial, Boschi extends the concept of the “structured whole”, now redefined as “whole” by German philosopher Georg Wilhelm Friedrich Hegel, by adding theragnostics as a peculiarity of radiopharmaceuticals (Boschi et al. 2024). The “whole” in Nuclear Medicine originated with the rise of [^18^F]FDG as a metabolic marker for most cancers, leading towards multiplicity of a range of metabolic tracers. By unifying thoughts, a shift was made from metabolic tracers towards radiopharmaceuticals targeting receptors expressed on tumor tissue opening the opportunity to combine diagnosis and therapy using the same (or very similar) molecules. The most recent discovery and use of FAPI in Nuclear Medicine by the group of Haberkorn (Loktev et al. 2018) adds to the “whole” and closes the ring to philosophy with “the success story of theragnostic agents originating in *academic* nuclear medicine” (Boschi et al. 2024).

### Is radiochemistry on the edge of a new renaissance?

#### By Sergio Todde

**Fig. 4:**
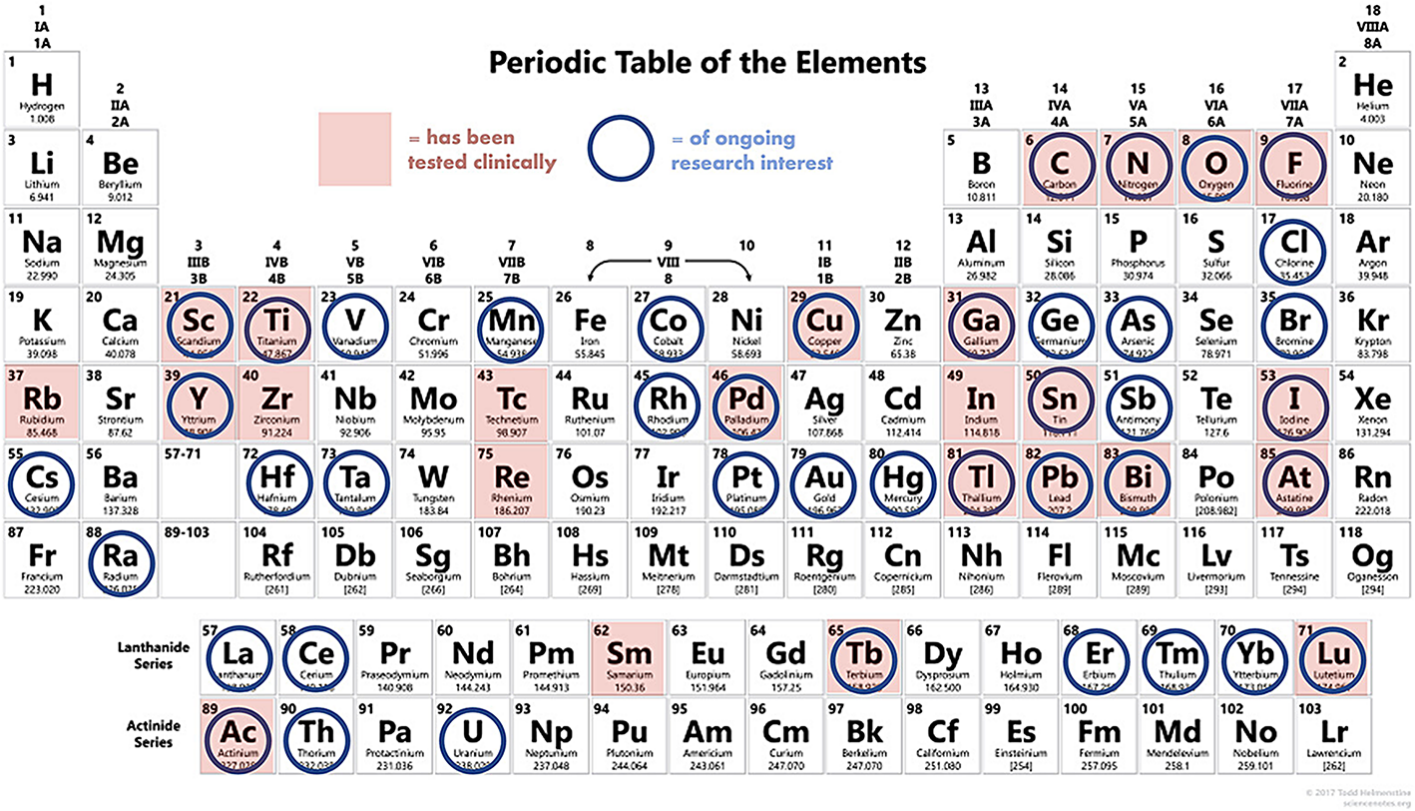
Summary of elements which possess radioisotopes of clinical and preclinical research interest for nuclear medicine, sometimes referred to as the “nuclear chocolate box of elements,” (Blower 2015)

The impressive growth of theranostics is fueling a renewed interest in radionuclide production and new radiopharmaceutical development. The selected paper (Bowden et al. 2023) considers both well-known and largely available radioisotopes such as fluorine-18 and carbon-11, as well as radionuclides that are currently of limited availability and/or whose chemistry has not been fully elucidated yet, like radium-223 or astatine-211, evaluating major challenges and opportunities, updating with recent advancements in their chemistry and trying to outline future perspectives (Fig. 4). For the above well-established radionuclides such as fluorine-18 and carbon-11, a great contribution in speeding up R&D is expected from the fast-evolving data-science assisted techniques (e.g. AI, machine learning), that will probably boost drug design and new radiopharmaceutical development. Moving to radiohalogens, bromine-76/77 might be a useful theranostic pair, but their production is still mostly limited by a troublesome targetry, while the alpha-emitter astatine-211 has a great potential in therapeutic application, but its chemistry is still not fully understood yet, and a weak point relies on its in vivo lability and redox sensitive nature. As for radionuclides to be used in coordination chemistry, there is a broad range of issues, some of which are quite common. For instance, their limited availability and the lack of stable isotopes that often make them understudied and difficult to characterize. From the “chemistry” point of view, the real challenge is in most cases to incorporate the radionuclides into suitable bifunctional chelators, to form in vivo stable complexes. To also mention opportunities, for several transition elements (e.g. Cu, Co), in vivo redox properties might be exploited to drive selective uptake or to predict their in vivo therapeutic behaviour.

### Using PARP inhibitors to bring Auger electrons near DNA for successful radiotherapy

#### By Xiang-Guo Li

The goal in nuclear medicine is to develop smart radiolabeled agents that can both identify what needs to be treated and treat what has been identified with high precision. This is known as radiotheranostics. Fluorine-18 is a popular radionuclide for clinical diagnosis with positron emission tomography (PET), and fluorine-18 can be replaced with another radionuclide, such as iodine-123, to transform an ^18^F-labeled small organic compound into a radiotheranostic agent due to the physical characteristics of iodine-123. This approach does not require a significant alteration of the chemical structure of the original radiopharmaceuticals to maintain similar pharmacokinetics, targeting affinity, and specificity between the pair of radiotheranostics. The ^123^I-labeled inhibitor of poly(adenosine diphosphate ribose) polymerase (PARP), [^123^I]CC1, was studied for the treatment of mice with PSN1 pancreatic adenocarcinoma, yielding promising results (Chan et al. 2023). [^123^I]CC1 is derived from the corresponding PET imaging agent [^18^F]olaparib (Wilson et al. 2019). Auger electrons only travel a few nanometers in vivo, but PARP inhibitors can reach close to DNA, making PARP inhibitors and Auger electron emitters well-suited for damaging DNA and causing tumor cell death. [^123^I]CC1 is an excellent example of this, and further results from clinical translation of [^123^I]CC1 would be highly anticipated.

### ^212^Pb-pretargeted theranostics for pancreatic Cancer

#### By Zhibo Liu

Lead-212 is considered to be an alpha therapeutic nuclide with broad application prospects due to its tumor-killing ability and moderate half-life. However, there are concerns of whether the high off-target toxicity of lead-212 may limit its clinical utility. Nevertheless, in 2023, an effective treatment of pancreatic ductal carcinoma (PDAC) was achieved by quickly and safely delivering lead-212 to tumors through a pretargeted strategy (Bauer et al. 2024). This work used trans-cyclooctene (TCO)-linked 5B1, an antibody targeting carbohydrate cell surface antigen 19 − 9 which is overexpressed on PDAC followed by an injection of ^212^Pb -labeled tetrazine, which binds to 5B1 in tumors. This strategy effectively combines long-lived antibodies and short-lived lead-212, achieving rapid enrichment of lead-212 and improving the effectiveness and safety of treatment. It is worth mentioning that this work also screened chelating agents based on charge, and found that DO3A with a slight negative charge has the best pharmacokinetic properties. In terms of safety, the authors also conducted dosimetry and histopathological studies and found that the release of the daughter nuclide bismuth-212 is still a problem unresolved. Although tumors can play a limited role in retaining bismuth-212, there is still free bismuth-212 metabolized by the kidneys, causing nephrotoxicity. Therefore, reducing the release of bismuth-212 is a problem that must be solved in the future application of lead-212.

### TAT alters the tumor microenvironment and induces systemic antitumoral immune responses

#### By Zhi Yang

Targeted alpha therapy (TAT) is a heavily investigated treatment modality. In order to study the immunomodulatory effect of TAT, actinium-225 labeled antihuCD20 sdAb 9079 ([^225^Ac]Ac-DOTA-9079) was prepared and the immunologic responses ensuing from [^225^Ac]Ac-DOTA-9079 TAT in human CD20 and ovalbumin expressing B16-melanoma model were studied (Ertveldt et al. 2023). Tumor growth was delayed and peripheral antitumoral T-cell responses were detected. At the tumor site, TAT modulated the tumor microenvironment (TME) with decreased protumoral alternatively activated macrophages and increased antitumoral macrophages and dendritic cells, as well as increased percentage of programmed death-ligand 1 (PD-L1)–positive (PD-L1^pos^) immune cells. Combination of TAT with PD-L1 blockade potentiated the therapeutic effect, but aggravated the adverse events. A long-term toxicity study revealed severe kidney damage ensuing from TAT. Although some strategies had been reported to decrease the kidney damage, further efforts should be warranted how to avoid adverse events. This study demonstrated that TAT can alter the TME and induces systemic antitumoral immune responses, which further showed the potential of TAT in tumor therapy.

### Astatine-211 production and logistics

#### By Nic Gillings

Astatine-211, a 100% alpha emitter with a half-life of 7.2 h, is considered to hold promise for radionuclide therapy applications, also known as targeted alpha therapy (Albertsson et al. 2022). Production of astatine-211 requires a cyclotron capable of producing an alpha beam of ca. 28 MeV, which is beyond the scope of most standard medical cyclotrons. Currently, there are only an estimated 10–15 production sites worldwide. As a consequence, the availability of this radionuclide is limited, which thereby limits the development and evaluation of astatine-211 labelled radiopharmaceuticals. Following cyclotron production, astatine-211 needs to be separated from the bismuth target material and provided in a chemical form compatible with radiolabeling procedures. This separation/purification is achievable by dry distillation or wet chemical separation.

A recent article (McIntosh et al. 2023), describes a new wet chemical procedure for purifying astatine-211 using a polystyrene-divinylbenzene chromatography resin. In this novel approach, astatine-211 is shipped to other sites in a dried resin column. Efficient recovery is achieved by elution with ethanol, yielding the radionuclide in a chemical form that was shown to be compatible with radiolabeling applications. So far, activities up to 2.2 GBq have been tested, with on-column holding times up to 34 h. This approach facilitates distribution to many sites over a large geographical area, where radiopharmaceutical development, preclinical and clinical trials may be performed.

### Microfluidic-based production of [^68^Ga]Ga-FAPI-46 and [^68^Ga]Ga-DOTA-TOC using the cassette-based iMiDEV™ microfluidic radiosynthesizer

#### By Emiliano Cazzola

**Fig. 5:**
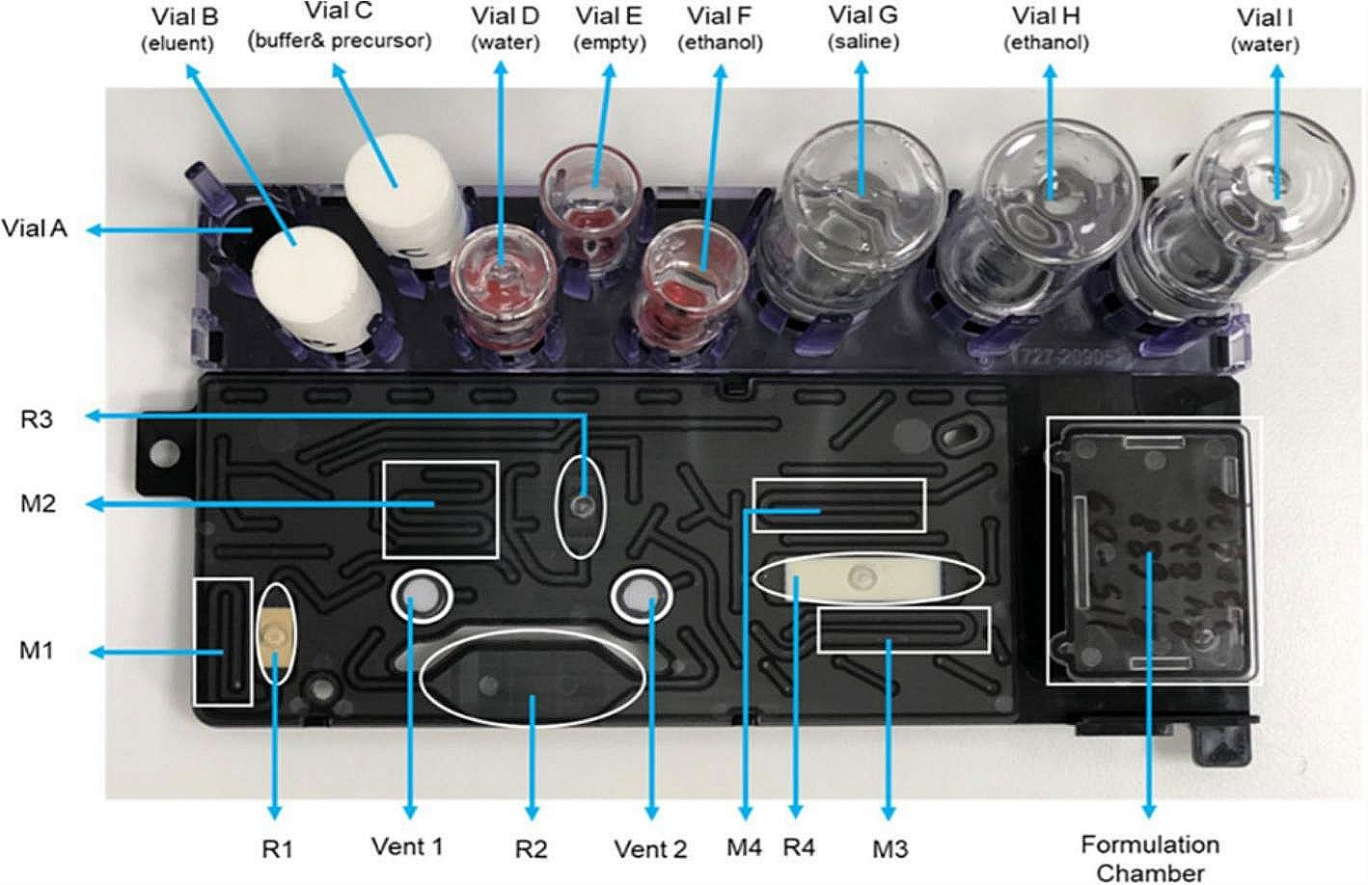
Picture of the microfluidic cassette as highlighted in Mallapura et al. 2023

A large number of new radiopharmaceuticals have become important for daily routines, and traditional production approaches, such as kit-based and synthesis modules, may not be sufficient to fulfill all the needs. Dose on demand, combined with radiometal precursors having low dilution volumes, is currently requested and present in the nuclear medicine field, but new approaches still need to be implemented. In a recent article, it was shown how to apply microfluidic techniques to tracer preparations using gallium-68 with a commercially available synthesis module and a single-use cassette (Fig. 5) (Mallapura et al. 2023). Both of these implementations are key factors in the application of new technology, contributing to operator dose reduction, enhanced reliability, reduced error rates, and the exclusion of cross-contaminations. By obtaining positive results, this paper shows the importance of initiating the tailoring of the radiosynthesis approach. It emphasizes the need to investigate appropriate reaction volumes and precursor amounts to enhance apparent molar activity. The reduction of the precursor amount is crucial not only in research scenarios, where limited material is commonly available for synthesis tuning and preclinical applications, but also in patient applications, thereby increasing target sensitivity. It is noteworthy that the quality control profile of the final formulations of the two radiopharmaceuticals presented is similar to those produced through traditional methods, all of which adhere to the general monograph on radiopharmaceutical preparations (European Pharmacopoeia 2023). Simultaneously, the authors address the challenges and future considerations essential for transferring this new approach and technology into routine applications.

### Zinc-mediated radiosynthesis of unprotected fluorine-18 labelled α-tertiary amides

#### By Wiktor Szymanski

**Fig. 6:**
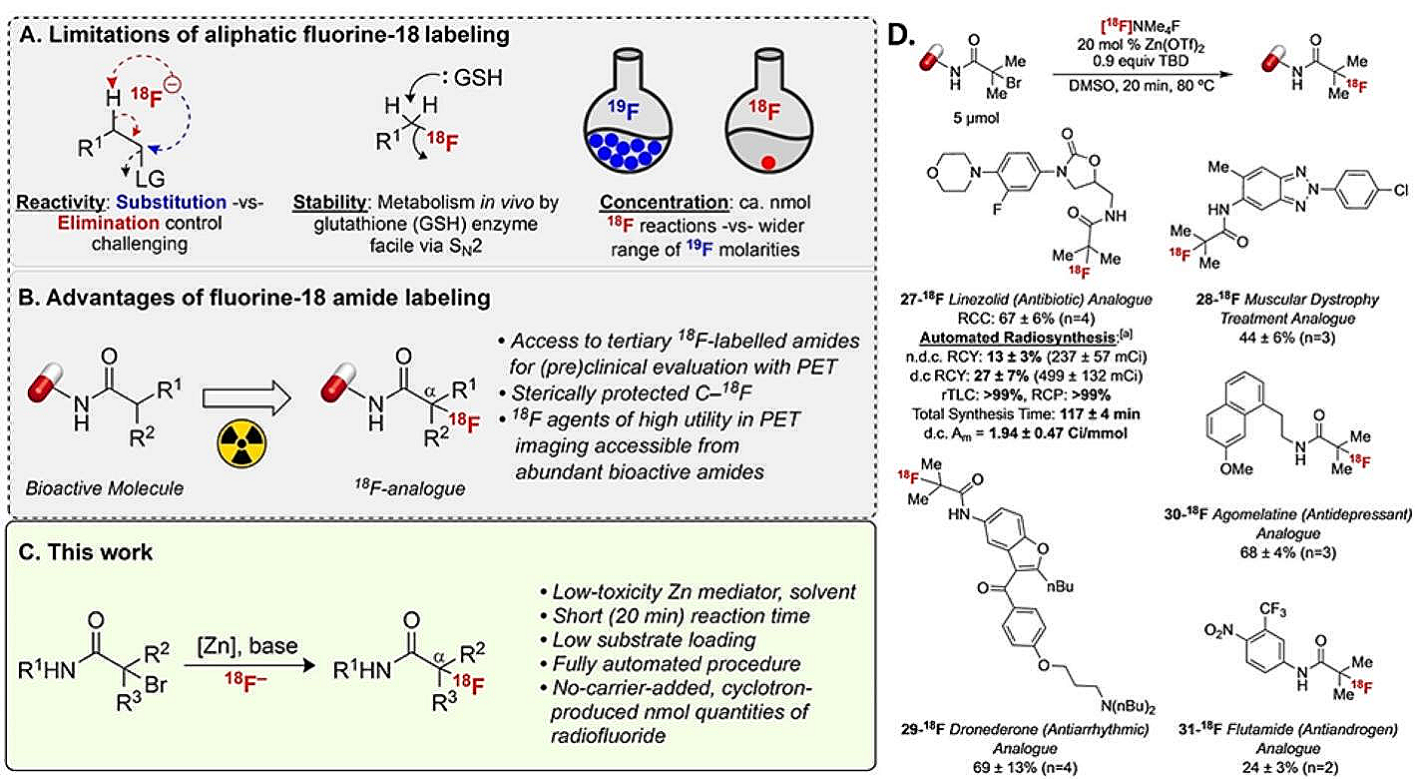
Fluorine-18 labeling of amides. (*A*) Limitations to the general aliphatic labelling with fluorine-18. (*B*) Advantages to using amides in fluorine-18 labeling for PET imaging. (*C*) The general scheme of the reaction enabled by the system (Wright et al., 2023) (*D*) Radiolabeling scope of drug-like molecules. Figure reproduced with permission from Wright et al. (2023)

More than a half of FDA-approved organic fluoride imaging agents are alkyl fluorides. The traditional S_N_2-reaction based synthesis of this class of compounds is still limited by competing processes (elimination), substrate structure (only primary and secondary electrophiles can be used as substrates), and the need for the use of protecting groups. The resulting primary and secondary [^18^F]fluorides can also be metabolically unstable (Fig. 6A).

A possible solution to these problems is the use of alpha-halo-amides as starting materials, which results in metabolically stable ^18^F-labeled amides, featuring a common motif in bioactive structures (Fig. 6B). The use of amides also gives access to strained cyclic intermediates of the process, which are derived by the transient nucleophilic substitution of a leaving group with the oxygen or the nitrogen of the amide bond.

In a recent publication, advantage of this reactivity was taken to establish a fast and mild method for introducing fluorine-18 to PET tracers (Wright et al. 2023) (Fig. 6C). The reaction uses zinc(II) triflate and acetonitrile as a solvent, and could be used to synthesize a library of ^18^F-labeled bioactive molecules with good to excellent radiochemical conversions (Fig. 6D).

### A further means of producing terbium sisters via alpha irradiation

#### By Nick Van Der Meulen

To date, there are very few means of being able to produce terbium radioisotopes such that they can be effectively used in preclinical research. Terbium-152, a PET radionuclide (t_1/2_ = 17.5 h), has been produced and collected via the Isotope Separation OnLine (ISOL) method, followed by chemical separation, to produce a radionuclidically pure product. Terbium-155, a SPECT radionuclide (T_1/2_ = 5.3 d) is produced in a similar manner, however, it has also been produced using enriched Gd target material via proton irradiation. A potential drawback of using protons, though, is the co-production of terbium-156 – a radioisotope of similar half-life to its terbium-155 sibling.

In recent work (Moiseeva et al. 2023), the authors propose a means of producing both radioisotopes via the alpha irradiation of enriched gadolinium-155 and europium-151 targets, respectively, in tandem. Should one irradiate the enriched Gd at alpha-beam energies of > 40 MeV, one can produce terbium-155 directly, as well as indirectly via the production of dysprosium-155 which, in turn, decays to terbium-155. A radionuclidic purity of > 99% can be obtained after 40 h of terbium-155 ingrowth from dysprosium-155.

The production of terbium-152 from its enriched europium-151 target occurs behind the Gd target in the beam, however, only 89% radionuclidic purity product is obtained, with terbium-151 (a partial alpha emitter) and terbium-153 as impurities.

The production of terbium-155 using the nuclear reaction proposed has potential towards preclinical applications, while the terbium-152 route proposed would require a dose assessment to ascertain its potential. It is clear that the medically-relevant terbium radioisotopes are sought after and it is encouraging to see options still being explored to produce it efficiently.

### Tandem isotope therapy with [^225^Ac]Ac-PSMA-617 and [^177^Lu]Lu-PSMA-617 in a murine model of prostate cancer

#### By Raymond M. Reilly

[^177^Lu]Lu-PSMA-617 (Pluvicto^®^, Novartis) is an effective β-particle-emitting treatment for metastatic castration-resistant prostate cancer (mCRPC) (Sartor et al. 2021). [^225^Ac]Ac -PSMA-617 emitting α-particles may also be effective for mCRPC (Kratochwil et al. 2018) but overcome resistance to [^177^Lu]PSMA-617 (Feuerecker et al. 2021). α-Particles emitted by actinium-225 have a short 50–100 μm range and high linear energy transfer (50–230 keV/mm) that is powerful for causing lethal DNA damage in cancer cells, while the maximum 2 mm range β-particles emitted by lutetium-177 have low LET (0.1-1 keV/mm) and are less damaging to DNA (Aghevlian et al. 2017). However, these advantages of actinium-225 may depend on tumour volume, since the range of α-particles is ideal for treating micrometer size lesions, while β-particles are more suited for treating millimeter sized tumours. [^225^Ac]Ac-PSMA-617 and [^177^Lu]Lu-PSMA-617 were compared alone or combined (i.e. tandem α- and β-particle therapy) for treating metastatic prostate cancer tumours in NSG mice (Meyer et al. 2023). An earlier study showed that at 3 weeks post-intracardiac inoculation of C4-2 prostate cancer cells, these mice had small volume metastatic disease (∼ 200 μm size lesions) while at 5 weeks, tumours were millimeter size (Stuparu et al. 2020). Mice were administered 40 kBq of [^225^Ac]Ac-PSMA-617 or 35 MBq of [^177^Lu]Lu-PSMA-617 to yield equivalent tumour absorbed doses. For tandem therapy, mice received 20 kBq of [^225^Ac]Ac-PSMA-617 and 17 MBq of [^177^Lu]Lu-PSMA-617 to minimize toxicity. In mice with small volume disease, [^225^Ac]Ac-PSMA-617 significantly inhibited tumour growth and prolonged survival vs. untreated mice, while [^177^Lu]Lu-PSMA-617 was ineffective. However, in mice with millimeter-sized tumours, both [^225^Ac]Ac-PSMA-617 and [^177^Lu]Lu-PSMA-617 inhibited tumour growth and improved survival. Nonetheless, [^225^Ac]Ac-PSMA-617 was the most effective, either alone or combined with [^177^Lu]Lu-PSMA-617. In conclusion, [^225^Ac]Ac-PSMA-617 effectively treated micrometer or millimeter sized lesions, while [^177^Lu]Lu-PSMA-617 was only effective for treating millimeter size tumours in preclinical mouse models of prostate cancer.

### Advances in the automated production of the glutamate-based tracer [^18^F]FSPG

#### By Carlotta Taddei

**Fig. 7:**
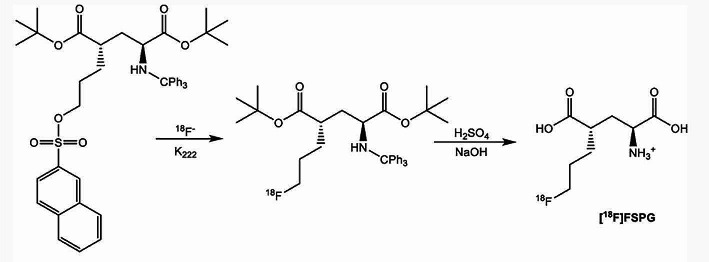
Reaction scheme for the synthesis of [^18^F]FSPG (Lin et al. 2023)

A recent publication (Lin et al. 2023) reported two automated procedures to produce the glutamate-based tracer (S)-4-(3-^18^ F-fluoropropyl)-L-glutamic acid ([^18^F]FSPG) with the aim to enhance the utility and clinical use of this radiotracer. [^18^F]FSPG is a PET tracer of recent imaging interest to target the cystine/glutamate antiporter (xc−), which is frequently overexpressed in cancer and several neurological disorders. In this study, [^18^F]FSPG was obtained using two commercially available radiosynthesizers within 45 min synthesis time in radiochemical yields (non-decay corrected) up to 30% and with starting activity in the range of 60–85 GBq (Fig. 7). Lin et al. implemented a simplified setup of solid-phase extraction (SPE) purification method with minimal processing time and SPE cartridge modification for both presented approaches. In addition, quality control tests were performed highlighting high radiochemical purity (> 95%) and no radiolysis was observed for a period of 8 h. Therefore, this work raises advances in the cGMP-compliant automated production of [^18^F]FSPG for clinical use.

### 7-[^18^F]Fluoro-8-azaisatoic anhydrides: versatile prosthetic groups for the preparation of PET Tracers

#### By Ralf Schirrmacher

**Fig. 8:**
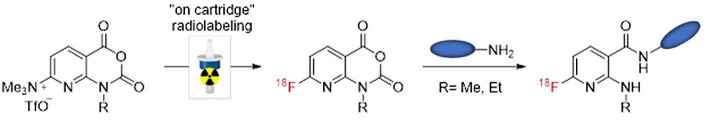
1-Alkylamino-7-[^18^F]fluoro-8-azaisatoic anhydrides ([^18^F]AFAs) as amine-reactive building blocks for click chemistry constitutes a new and promising entry to prosthetic group radiolabeling (Gröner et al. 2023)

It seems that the development of new prosthetic groups (PGs) is not coming to an end soon. Recently it was demonstrated that prosthetic group chemistry is still alive and contributing to the field of radiochemistry. Introducing 1-alkylamino-7-[^18^F]fluoro-8-azaisatoic anhydrides (^18^F-AFAs) as amine-reactive building blocks for click chemistry constitutes a new and promising entry to PG radiolabeling (Fig. 8) (Gröner et al. 2023). One highlight characterizing the use of ^18^F-AFAs is the ability to conveniently prepare them “on cartridge” via ^18^F-fluorination of cartridge-bound trimethylammonium precursors. Amines readily react with ^18^F-AFAs via formation of chemically stable amide bonds. Furthermore, the AFA structure lends itself towards N-functionalization giving access to more versatile building blocks including azides and alkynes for triazole formation, one of the most popular click chemistries in organic and radiochemistry. To prove general applicability, ^18^F-AFAs were applied to the preparation of ^18^F-labeled PSMA ligands in good radiochemical yields demonstrating excellent in vivo stability and favorable visualization of small PSMA-positive tumor lesions.

### A novel small-molecule tracer for α-synuclein PET imaging: a promising leap in neurodegenerative disease diagnostics

#### By Zijing Li

The development of specific PET tracers for disorders related to α-synuclein (α-syn), such as Parkinson’s disease, has faced various challenges including the relatively low abundance of α-syn in the brain, structural similarity, and coexistence with other proteins like amyloid-beta (Aβ) and tau (Korat et al. 2021). Therefore, there has been a persistent outstanding request for a dedicated PET tracer.

With this opportunity, the research article published in Cell (Xiang et al. 2023), introduces the development of a novel small-molecule PET tracer named [^18^F]F0502B. With high affinity and selectivity for α-syn over Aβ and tau proteins (α-syn fibrils K_D_: 10.97 nM, Aβ fibrils K_D_: 109.2 nM, tau fibrils K_D_: 120.5 nM), a significant advancement, [^18^F]F0502B enables more precise detection of α-synuclein aggregates, a crucial pathological hallmark of synucleinopathies. This capability allows for a clearer differentiation between synucleinopathies and other neurodegenerative diseases such as Alzheimer’s disease, which is crucial in clinical settings for accurate diagnosis and treatment planning. The structure of the complex formed by F0502B and α-synuclein fibril, revealed through cryo-electron microscopy, offered a profound insight into the binding mechanism. The results of PET imaging in non-human primate models of Parkinson’s disease demonstrated the efficacy of [^18^F]F0502B in detecting α-synuclein aggregates within the living brain.

The translation of this discovery into clinical practice has the potential to enhance more accurate and earlier detection of α-synuclein-related neurodegenerative diseases. This is particularly crucial in distinguishing these diseases from other neurodegenerative disorders, thereby facilitating better disease management and improving the quality of life for patients affected by these conditions.

### Is the African radiopharmacy being triggered towards its optimistic developmental move?

#### By Yohannes Jorge Lagebo

Radiopharmacy (RP) is the professional discipline dealing with the comprehensive aspects of radiopharmaceuticals (RPs) and RPs in turn are the vital components of actualizing patient nuclear medicine diagnostic and therapeutic procedures. Without RPs/radionuclides the NM patient procedures are entirely impossible. The RP/nuclear medicine science and technology has significantly advanced globally in the last over four decades till present in order to be able to resume its more impacting role in serving patients. However, this has not been the practical reality in Africa. Consequently, patients in the majority of countries within this continent have been the most underserved in RP/nuclear medicine services. The related study by (Duatti et al., 2013) confirmed this reality during its mentioned study period.

Another survey-based study (Ekoume et al. 2022) witnessed this reality as well.The parameters employed by this group to evaluate the deficiencies in radiopharmacy developments and practices within the continent were the extent of human resource development in radiopharmacy: available facilities/equipment, the types of RPs/radionuclides used for patients, ability to manufacture various RPs/radionuclides and the scope of radiopharmacy practices.This group identified significant gaps and clear deficiencies against the indicated parameters which were more peculiar to this continent. This study has also indicated the currently triggered positive RP/nuclear medicine developmental initiatives underway and the anticipated glimpse of hope in most parts of Africa with the associated efforts. Despite the fact that there have been promising activities on being initiated to date in the radiopharmaceutical developments in Africa (Jalilian et al. 2023), the issue generally is not yet still to be meaningfully translated into practice within the continent attributed to its slow/stagnated development of the indicated contemporary science and technology in the continent.

In the other most recent African focused associated study as well (Kleynhans et al. 2023) similar results were observed too. However, the study also showed that this situation is not uniform throughout the continent and there are disparities. In this regard very few countries in the northern and southern parts of the continent comparatively are in the better developmental scenario. Besides the study also indicated that some encouraging efforts are underway and visible outcomes are being observed currently in radiopharmaceutical developments in the broader coverage of the continent versus the above indicated developmental parameters. Accordingly, we may conclude this review by raising the question “Is the African Radiopharmacy heading towards its optimistic developmental move?”

### Trends in Radium-223 use in personalized radionuclide therapy: current challenges and future perspectives

#### By Naoual Bentaleb

Radium-223 is a short-lived (11.4 d) alpha emitter with attractive nuclear properties for use in personalized radionuclide therapy. Due to the success of Xofigo® (radium-223 dichloride) that is the first targeted alpha therapy (TAT) approved for the palliative treatment of bone metastases, several new prospective clinical ^223^Ra-based applications are currently eliciting considerable research interest.

A comprehensive overview on the multiple research novelties regarding the use of radium radioisotopes in personalized radionuclide therapy of various cancer types was recently presented, with focus on radium-223 (Franchi et al. 2023). The authors found that the most important challenge remains the development of constructs capable of incorporating radium radioisotopes into cancer-targeting drugs in a way to transport it to the target tissue and to prevent uncontrolled redistribution as no optimal vector has been found to date with sufficient stability for clinical use.

Interestingly, this paper describes the chemical characteristics of two possible approaches to bind radium radioisotopes in a carrier system. The first one is to use suitable bifunctional chelator in regard to the coordination chemistry of radium ions. The encapsulation of radium radioisotopes in nanocarriers is also an option prompted to bind radium ions.

The potential possibility to use radium-223 in imaging and the applicability of the pair barium- 131/radium-223 as a theranostic pair was also discussed by Franchi and colleagues as the coordination chemistry of barium ions is similar to that of radium ions.

### Advancements in alpha therapy: the role of micellar [^223^Ra]RaCl_2_ in targeting osteosarcoma with improved pharmacokinetics and biochemical safety

#### By Marta De Souza Albernaz

The article titled “Micellar Solution of [^223^Ra]RaCl_2_: Reaching renal excretion Potent Efficacy in Osteoblastic Osteosarcoma in PDX Model Biochemistry Alterations and Pharmacokinetics” (Pijeira et al. 2024) presents a comprehensive study on the use of a nano-sized micellar solution of radium-223 dichloride ([^223^Ra]RaCl_2_) for targeted alpha therapy in oncology, specifically addressing its biodistribution, pharmacokinetics, and effect on osteosarcoma in a patient-derived xenograft (PDX) model. Radium-223 is an alpha-emitter radiopharmaceutical previously used for treating castration-resistant prostate cancer with bone metastases. However, it caused short-term side effects like diarrhea and vomiting due to gastrointestinal excretion. The study aimed to overcome these limitations by using a nanosized micellar solution of [^223^Ra]RaCl_2_. The micellar [^223^Ra]RaCl_2_ was prepared by adding [^223^Ra]RaCl_2_ aqueous solution to a Pluronic F127 micellar dispersion. Its mean particle size was around 149 nm, and it was characterized using dynamic light scattering. In vivo studies in healthy mice showed that the micellar [^223^Ra]RaCl_2_ had high bone-targeting properties and was primarily excreted through the kidneys, contrasting with the non-micellar form’s gastrointestinal excretion. Pharmacokinetic analysis revealed low distribution volume and a longer elimination half-life. Biochemical analysis indicated that the micellar [^223^Ra]RaCl_2_ did not induce significant changes in various enzymatic activities in treated mice. In osteosarcoma treatment, the micellar form showed regression of tumors with substantial areas of necrosis in the treated group. The study highlighted the potential of micellar suspensions as nanocarriers to improve drug delivery, reduce side effects, and enhance the therapeutic index in cancer treatment. The renal excretion pathway of the micellar solution could minimize gastrointestinal side effects, and its bone-seeking properties were retained in the new formulation. The findings demonstrated the efficacy and safety of the micellar [^223^Ra]RaCl_2_ in reducing gastrointestinal excretion, promoting renal excretion, and effectively regressing osteosarcoma. However, the authors recommended further studies for therapy efficacy assessment and clinical translation, including dose-response outcomes and organ/tissue dosimetry. The article provides a detailed examination of the potential benefits of using a nanomicellar formulation of [^223^Ra]RaCl_2_ in oncology, particularly for osteosarcoma treatment, highlighting its improved safety profile and effective targeting properties.

### ^134^Ce/^134^La: potential allies for ^225^Ac in the theranostic arena

#### By Suzanne Lapi

The development of ^225^Ac-radiopharmaceuticals has become an area of intense interest for the radiopharmaceutical community. However, a lack of closely matched suitable PET imaging radionuclides has impeded the development of closely aligned theranostic strategies for this isotope. Therefore, an investigation of the ^134^Ce/^134^La generator as an imaging strategy for actinium-225 has been reported (Bobba et al. 2023). Cerium-134 decays by electron capture with a 3.2 d half-life to the positron emitter lanthanum-134 with a half-life of 6.45 min. The authors leverage the half-life and chemical characteristics of cerium-134 (and chemical similarity to actinium-225) combined with the physical decay characteristics of lanthanum-134 to enable a new theranostic strategy.

In this recent exciting work, cerium-134 was used to radiolabel the DOTA and MACROPA chelators and in vivo studies were conducted with radiolabeled PSMA-617 and an YS5 antibody in tumor bearing mice. The authors found superior labeling with MACROPA constructs and very similar distributions of the radiolabeled actinium-225 and cerium-134 chelators illustrating (bio)chemical similarity between the two complexes. The authors also observed evidence of release of the lanthanum-134 from the chelator. Studies in animals bearing prostate cancer xenografts illustrated a close alignment of the ^134^Ce- and ^225^Ac-MACROPA-PEG4-YS5 complexes with the exception of the liver and spleen. The authors conclude that while differences exist in the biodistributions of these compounds likely due to the release of the lanthanum-134 after the decay of cerium-134, further studies are warranted with the ^134^Ce/^134^La in vivo generator and that ^134^Ce-radiopharmaceuticals may play an important role in the development of ^225^Ac-radiopharmaceuticals.

### Chelating [^227^Th]Th^4+^/[^89^Zr]Zr^4+^: efforts towards the expansion of the theranostic ‘tool-box’

#### By Caterina Ramogida

Back when I was an impressionable graduate student attending an international conference on radiopharmaceuticals, I recall one of the speakers presenting their work on the production of gallium-68 for PET. During their talk, they addressed the audience about the prevailing tendency in our field to possess a short attention span – swiftly moving on to the next ‘radionuclide of interest’ without fully translating the research of the previously identified ‘next best nuclide’. Although I respected and understood their sentiment, it was also discouraging for me, as a young scientist, to absorb this mindset. The diversity of research, particularly in the realm of radiometal chemistry, has always been and continues to be captivating to me. The ability to tailor the choice of radionuclide for a specific purpose, be it imaging, therapy, or a combination of both for theranostics, gives the field great promise to make advances in personalised medicine.

The work presented in the paper entitled “Macrocyclic 1,2-Hydroxypyridinone-Based Chelators as Potential Ligands for Thorium-227 and Zirconium-89 Radiopharmaceuticals” (Woods et al. 2023) is a great example of the surge in interest in developing the next generation of theranostic radiopharmaceuticals using “exotic” or less “mainstream” radiometals. Thorium-227 ([^227^Th]Th^4+^) emits five alpha particles through its decay to stable lead-207, this potent alpha emitter has shown (pre-) clinical promise for use in radiopharmaceutical therapy. Notwithstanding their disparate ionic radii, zirconium-89 ([^89^Zr]Zr^4+^) shares similar coordination chemistry to [^227^Th]Th^4+^ and may form a suitable theranostic pair allowing PET imaging with the former. Using a theranostic pair of similar coordination chemistry would allow the same bifunctional chelator (BFC) to be employed. With this in mind, the authors developed and studied two non-bifunctional cyclen-based 1,2-hydroxypyridinone (HOPO) chelators, given the tendency of HOPO to form stable complexes with + 4 metal ions. The chelators exhibited excellent ability to incorporate [^227^Th]Th^4+^ and [^89^Zr]Zr^4+^ quickly (30 min) at ambient temperature. Both sets of radiometal-complexes remained inert when challenged against serum in vitro. These scaffolds are promising architectures for incorporation into a theranostic radiopharmaceutical using [^227^Th]Th^4+^ and [^89^Zr]Zr^4+^, as such the preparation of bifunctional analogues are warranted to further study their applicability.

In summary, there is ample space for all radionuclide research in our field as we endeavour to cultivate a diverse and inclusive environment, both in the way we conduct research and assemble our teams.

### Imaging cell death: status and way forward

#### By Archana Mukherjee

Imaging cell death holds immense potential in oncology, enabling the evaluation of treatment responses for tailored strategies. Its applications further extend to cardiology and neurology, addressing specific pathological conditions. A recent review consolidates various strategies and presents the status of radiopharmaceuticals investigated thus far for imaging cell death (Ho Shon and Hogg 2023). The Nomenclature Committee on Cell Death, 2018 has categorized cell death into distinct types which offers valuable insights into the molecular mechanisms of cell death, paving the way for the development of tracers that target specific pathways or biomolecules. A list of radiotracers developed so far targeting the externalization of phosphatidylserine (PS) and phosphatidylethanolamine (PE), caspase activation, and La autoantigen is presented with stages of development. The most extensively studied in preclinical models and clinical trials, radiolabeled Annexin V and its derivatives, have shown limitations in biodistribution and are yet not being used in clinical practice (Ho Shon and Hogg 2023). However, in a clinical setting to assess response to antitumor therapy, a probe giving a strong signal for cell death is more appropriate compared to a pathway-specific radiotracer (Rybczynska et al. 2018). Therefore, rather than focusing on pathways, there is potential for targeting cell phenotypes. Among them, radiotracers that assess the loss of plasma membrane integrity have shown more promising results compared to those targeting mitochondrial membrane potential and cell membrane acidification.

Cell responses differ following various therapeutic interventions, across different tumor types, and due to tumor heterogeneity. Given the dynamic nature of this process, determining the optimal timing for quantifying treatment response in terms of effective cell death is a significant challenge. Finally, the authors recommend a detailed evaluation of promising radiotracers for cell death imaging to optimize timing for quantifying treatment response (Ho Shon I and Hogg PJ, 2023). Additionally, multicentre clinical trials to establish the correlation between cell death imaging and subsequent clinical responses should be conducted to achieve the aim of developing an ideal radiotracer for cell death imaging.

### Let PET be multichromatic! Simultaneous quantitative imaging of two PET radiotracers

#### By Javier Ajenjo

Multiplexed Positron Emission Tomography (mPET) serves as an advanced reconstruction technique, skillfully segregating two PET isotopes within a single acquisition based solely on their distinct gamma emissions (Pratt et al. 2023). The incorporation of a second tracer in mPET not only imparts additional temporal insights but also promises enhanced visualization when synchronized with molecular imaging agents. The paper describing works by the teams of Grimm and Herraiz demonstrate the seamless adaptation of mPET to both preclinical and clinical PET/CT systems, eliminating the need for energy windowing while preserving PET’s quantitative prowess. The versatility of mPET is exemplified through its application in exploring drug-loading dynamics, binary distributions, and concurrent quantification of dual immune markers. For example, by assessing the in vivo biodistribution of intravenously administered [^124^I]I-trametinib and [^18^F]FDG, [^124^I]I-trametinib alongside its nanoparticle carrier [^89^Zr]Zr-ferumoxytol, or the interaction between prostate-specific membrane antigen (PSMA) and antigen receptor T-cells, employing [^68^Ga]Ga-PSMA-11 and [^124^I]I. As a reconstruction method, mPET holds the potential to augment information density derived from a single PET imaging session. Anticipating future developments in energy discrimination, mPET stands poised to usher in an era of multi-color PET imaging, offering images of unprecedented richness and surpassing the current limitations of PET in research and clinical applications.

### Peptides as radiopharmaceuticals - challenging but feasible

#### By Winnie Deuther-Conrad

Peptide hormones activate receptors at the cell membrane, resulting in G-protein-mediated activation of a complex system of signaling cascades that initiate the intracellular biological response. The receptor affinity is high for all hormones, enabling an effect in the presence of physiological concentrations of the peptide hormone. The development of radiation-emitting hormone analogues as imaging agents for receptor-related diseases is of great interest. However, unmodified linear peptides have a significant issue due to their short biological half-life caused by rapid proteolysis in plasma. Therefore, modifications to the peptides are necessary to increase their stability and prolong their half-life. The 28-amino acid peptide hormone ghrelin and its receptor, the growth hormone secretagogue receptor (GHSR), play a crucial role in regulating appetite and are therefore relevant for cancer-associated anorexia and cachexia. However, noninvasive imaging and pharmacological modulation of GHSR1a remain significant challenges. A library of structural analogues of ghrelin was synthesized (Childs et al. 2023). Starting with a previously identified ghrelin-based analogue with subnanomolar affinity but unfavourable pharmacokinetics in mice as a starting point, the authors conducted a comprehensive study of the structure-activity-stability relationship to identify a candidate with suitable stability, affinity, and potency (Charron et al. 2017). After identifying the metabolic weak point between positions Leu^5^ and Ser^6^ in ghrelin, which caused the initial analogue to degrade in vitro due to serum and liver enzymes, a targeted library of analogues was developed. This was achieved by substituting amino acids such as alanine, D-amino acids, or non-canonical amino acids at positions 5 and/or 6. Additionally, the peptide backbone was modified and the impact of local chemical space, flexibility, and chemical properties on binding affinity, serum stability, and liver stability was thoroughly analysed. Subsequently, the ability of the new analogues to activate GHSR1a-related signaling pathways was investigated through various functional experiments, including calcium signaling and BRET assay. Overall, it remains to be determined whether the advantageous properties of the new ghrelin-based analogues will also be observed in vivo. Overall, although it remains to be determined whether the advantageous properties of the new ghrelin-based analogues will also be observed in vivo, the systematic and comprehensive experimental work is of general significance for the development of radio-peptide theranostics that surpass ghrelin analogues (Childs et al. 2023).

### Marshalling the potential of auger electron radiopharmaceutical therapy

#### By Cécile Bourdeau

Radiopharmaceutical therapy is emerging as a safe and effective targeted approach to treating many types of cancer. In radiopharmaceutical therapy, radiation is systemically or locally delivered using pharmaceuticals that either bind preferentially to cancer cells or accumulate by physiological mechanisms. In this field Auger electron radiopharmaceutical therapy may have the same therapeautic efficacy as α particles for oncologic small disease with lower risks of normal-tissue toxicity.

The cited authors present the potential of Auger electron radiopharmaceutical therapy to enhance and impact future efforts in Auger electron radiopharmaceutical therapy research. They selected a list of well-studied and emerging Auger electron-emitting radionuclides. The selection process for preferred Auger electron-emitting radionuclides favored different parameters each studied and described in the article (Bolcaen et al. 2023).

First of all, those with a higher number of Auger electrons emitted (an average Auger electron yield of 20 or more per decay is preferred for Auger electron radiopharmaceutical therapy), preferred half-life (to take account of all manufacturing, transport, pharmacokinetic and other constraints), current worldwide availability (benefit from the distributed global small- to medium-sized cyclotrons (30 MeV), target availability, ease of radiochemical separation (most of the promising Auger electron-emitting radionuclides discussed in the article lack efficient radiochemical purification), chelator availability, molar activity (high molar activity is very important as it enables a maximum number of radioactive atoms to be delivered to its target site and reach optimal therapeutic efficacy), vector availability, and overall dosimetry score (the development of standardized dosimetry practices is necessary for Auger electron radiopharmaceutical therapy. Overall the work provides a nice overview and recommendations in Auger electron radiopharmaceutical therapy research.

## Conclusions

Trends in radiochemistry and radiopharmacy are highlighted. Hot topics cover the entire scope of EJNMMI Radiopharmacy and Chemistry, demonstrating the progress in the research field in many aspects.

## Data Availability

Datasets mentioned in this article can be found in the cited articles.

## Abbreviations

AFA: 1-alkylamino-7-[^18^F]fluoro-8-azaisatoic anhydrides
BRET: bioluminescence resonance energy transfer
DNA: deoxy nucleic acids
EPR: enhanced permeability and retention
FAPI: fibroblast activating protein inhibitor
FDA: Food and Drug Agency
FDG: 2-[^18^F]fluoro-2-deoxyglucose
GHSR: growth hormone secretagogue receptor
LAFOV: Long axial Field-of-View
LET: linear energy transfer
NSG: NOD scid gamma
PARP: poly(adenosine diphosphate ribose) polymerase
PDX: patient derived xenograft
PET: positron emission tomography
PSMA: prostate specific membrane antigen
RP: radiopharmaceutical
SPECT: single photon emission computed tomography
TAT: targeted alpha therapy
